# Thai version of shortened Oral Impact on Daily Performances index for evaluating oral lichen planus patients

**DOI:** 10.1186/s12903-023-03094-9

**Published:** 2023-06-12

**Authors:** Sasirin Yiemstan, Pornpan Piboonratanakit, Sudaduang Krisdapong

**Affiliations:** 1 Support Service Department, Royal Thai Armed Forces Headquarters, Bangkok, 10210 Thailand; 2 Department of Oral Medicine, Chulalongkorn University, Bangkok, 10330 Thailand; 3 Research Unit in Oral Diseases, Chulalongkorn University, Bangkok, 10330 Thailand; 4 Dental Public Health Researcher (Freelance), Bangkok, Thailand

**Keywords:** Oral lichen planus, OLP, Shortened version of Oral Impact on Daily Performances, Shortened version of the OIDP index

## Abstract

**Background:**

Patients’ perceptions and Oral Health-related Quality of life (OHRQoL) are important parts of dental treatment in all fields, including oral lichen planus (OLP) patients. A shortened version of the Oral Impact on Daily Performances (OIDP) might be more practical and feasible in clinical setting due to the busy nature of oral medicine clinics and staff availability to conduct the interview for data collection. The aim of the study was to develop a Thai version of shortened OIDP for assessing the OHRQoL in OLP patients.

**Methods:**

Two types of shortened OIDP versions were tested in 69 OLP patients, one comprising the most commonly interfered with daily performances (OIDP-3 and OIDP-2) and another comprising either the highest frequency (OIDP frequency) or severity score (OIDP severity). The Numeric Rating Scale (NRS) and Thongprasom sign score were used to assess oral pain and clinical severity. Spearman rank-order correlation coefficients (r_s_) were used to demonstrate the associations between the shortened and original OIDP, pain, and clinical severity.

**Results:**

OIDP-3 (Eating, Cleaning, and Emotional stability) and OIDP-2 (Eating and Emotional stability) were developed. The associations of the original OIDP with OIDP-3 and OIDP-2 (r_s_ = 0.965 and 0.911) were significantly higher than those of the original OIDP with OIDP frequency and OIDP severity (r_s_ = 0.768 and 0.880). The original OIDP, OIDP-3, and OIDP-2 were more significantly associated with pain compared with OIDP frequency and OIDP severity. The association between the clinical severity and oral impacts assessed by the original OIDP, OIDP-3, and OIDP-2 were similar and had higher correlation coefficients compared with those of OIDP frequency and OIDP severity.

**Conclusion:**

OIDP-3 and OIDP-2 performed more similarly to the original OIDP than OIDP frequency and OIDP severity in assessing the OHRQoL of OLP patients.

**Trial registration:**

The trial was registered at the Thai Clinical Trials Registry (TCTR identifier: TCTR 20190828002).

## Background

Oral lichen planus (OLP) is a chronic inflammatory oral disease whose symptoms vary from a mild burning sensation to severe pain [1]. In the OLP literature, pain perception is used as a primary measure and a proxy for patient-based outcomes [2, 3]. Various pain measurement tools have demonstrated good correlations with the clinical severity of OLP lesions, such as the Numeric Rating Scale (NRS), McGill pain questionnaire, Visual Analogue Scale (VAS), and Change in Symptom Scale (CSS) [3, 4]. Patient-centered approaches have used the Oral Health-related Quality of Life (OHRQoL) measures [5]. Most studies on the OHRQoL of OLP patients used a generic measure, the Oral Health Impact Profile (OHIP), while some used a specific questionnaire designed for chronic oral mucosal diseases, the Chronic Oral Mucosal Disease Quality of Life index (COMDQ) [4, 6–9]. However, the required cross-cultural adaption and validation processes limit the use of these indexes in Thailand. In our study, the Oral Impact on Daily Performances (OIDP) index was chosen because the index was developed and systematically validated in all age groups of the Thai population, i.e., primary school children, teenagers, adults, and elderly [10–14]. In addition, the OIDP was used as a part of 6^th^ to the present (9^th^) Thailand National Oral Health Surveys [12, 15] and has been found to be a valid measure for use in many clinical trials, including patients with a prosthesis, dental implants, cleft lip and palate, and OLP [16–20]. Moreover, the conceptual framework of the OIDP is different from the OHIP index because the OIDP assesses only the ultimate impacts, that are, difficulties in daily life performances which are the consequences of intermediate impacts of pain, discomfort, functional limitation and change in appearance [11]. The OIDP considers the assessment of intermediate impacts as repetitive. However, this consequential issue is not taken into consideration of OHIP. In addition, pain measurement is already a standard patient-based outcome in OLP patients and was included as another measure in our study.

The OIDP index assesses oral impacts during the past six months on 8 daily performances and focuses on the change in behaviors in daily life, which is the ultimate impact according to its conceptual framework [11]. The method of collecting the OIDP data is based on the frequency and the severity of the impacts on each of the eight performances, resulting in 16 answers for each patient [11]. Therefore, some studies reduced the time-consuming data collecting procedure by using only the frequency or the severity score [11, 21–27]. The OIDP frequency scale is a valid shortened version for use in Tanzania, Uganda, India, Sudan, Nigeria, Norway, and Sweden [21–26]. The advantage of using the frequency score rather than the severity score is the objectivity of the patient’s answers. However, some studies considered personal feelings, although subjective, as crucial to a patient’s perceived problems. In these cases, the severity score was used [27].

A previous study in Thai patients demonstrated that the OIDP was a valid and reliable instrument to use with OLP patients. It was well associated with the oral pain and the clinical severity of OLP, indicating the criterion and construct validity of the index, respectively [20]. Furthermore, the OIDP differentiated between the clinical severity differences in OLP, indicating the discriminative ability of the index. However, the time-consuming interview process limits the practicality of using the OIDP. A shortened version that compromises on the comprehensive understanding of a patient’s OHRQoL, while retaining its discriminative ability would be an alternative tool for integrating the OHRQoL concept into the routine treatment of OLP patients.

The objectives of this study were, first, to assess the clinical characteristics and OHRQoL of OLP patients using the OIDP index. Second, Thai version of shortened OIDP index was developed, and its association with the original OIDP, oral pain, and the clinical severity of OLP were determined. In addition, the discriminative ability of the shortened versions was compared with that of the original version.

## Methods

### Study participants

The source data of this study was from our previous study investigating the association between clinical signs of OLP and OHRQoL [20]. The sample size calculation was performed with G-power software. Proportions of oral impacts experienced by the patients with OLP and with aphthous lesions reported by a previous similar study [28] were used to calculate the sample size. An estimated sample size of 64 was obtained and using 10% over-sampling resulted in a sample size of 71 patients. The eligibility criteria comprised newly diagnosed or treated patients with a clinical and biopsy-based diagnosis of OLP or compatible with OLP [29], being 18 years or older, presence of OLP lesions, able to communicate, and provide informed consent. Subjects were excluded if they had a current active infection of dental caries, periodontal disease, or acute illness of any kind including temporomandibular disorder or presence of any other oral mucosal lesions because subjects might not be able to differentiate whether their perceived problems were the results of OLP or not. Pregnancy and smokers were also excluded.

### Data collection

The data were collected by one well-trained and calibrated examiner. Ten patients were selected for evaluation and the examiner’s results were calibrated against an expert in using the OIDP index [12]. The Inter-examiner reliability demonstrated high reliability with an intraclass correlation coefficient (ICC) of 0.910 (*P* < 0.001). In addition, 10% of the patients were re-examined and re-interviewed to determine the intra-examiner reliability, which exhibited very good agreement (ICC = 0.911, *P* < 0.001). The data collected consisted of the patients’ demographic information, clinical characteristics, and patients’ perceptions. The demographic data comprised sex, age and systemic conditions. The clinical characteristics comprised the duration since the first diagnosis that were obtained from dental charts, OLP localization, and number of OLP affected sides of which the maximum was 11 sides (right and left buccal mucosa, dorsal and ventral tongue, upper and lower lips, upper and lower gingiva, palate, floor of the mouth, and soft palate). Furthermore, the severity of the OLP lesions was measured using the Thongprasom sign scoring system that categorized a lesion into 5 levels; score 1: mild white striae only, score 2: white striae with an atrophic area less than 1 cm^2^, score 3: white striae with an atrophic area equal to or greater than 1 cm^2^, score 4: white striae with an erosive area less than 1 cm^2^, score 5: white striae with an erosive area equal to or greater than 1 cm^2^. For patients with multiple lesions, the Thongprasom sign score was recorded for the most severe lesion [30].

The patient’s perceptions comprising pain and OHRQoL were also determined. For pain perception, the patients were asked to rate their current pain intensity on a 0–10 NRS scale, 0 for no pain at all and 10 for the worst imaginable pain. In addition, the OHRQoL was assessed with the individual interviewing method using a Thai version of the OIDP index [11]. The OIDP index was designed to assess OHRQoL of subjects during the past 6 months. However, we focused on the difficulties of which patient’s perceived cause was OLP lesions, rather than overall oral problems. For example, we excluded the answer if patient perceived other oral diseases such as dental caries as the cause of problems. If a patient reported having any OLP-related difficulty on a performance, the frequency and severity scores for that performance were recorded. The frequency score, ranging from 0–5, was assessed through the question “During the past six months how often have you had this difficulty? Have you had this difficulty on a regular basis or only occasionally?”. For a chronic pattern of oral impacts through which patients experienced difficulty on a regular basis during the past 6 months; score 0, no difficulty; score 1, less than once a month; score 2, once or twice a month; score 3, once or twice a week; score 4, three to four times a week; and score 5, every or nearly every day. In case of oral impacts experienced occasionally, the frequency was scored by the number of days that the patient had difficulty; score 1, 1–5 days; score 2, 6–15 days; score 3, 16–30 days; score 4, 1–3 months; and score 5, more than 3 months. If a frequency score was between 1–5, the participant was asked how severe the effect of the difficulty was on their daily life. The severity score ranged from 0–5; 0, never affected; 1, very little; 2, little; 3, moderate; 4, severe; and 5, very severe.

### Data analysis

Statistical analyses were performed using SPSS for Windows, version 22.0. The outcomes of the OIDP index were calculated as extent and intensity [12] (Table 1). Extent refers to the number of performances with impacts (range from 0–8) representing how extensive the oral impacts on daily performances were. For each performance, a performance score was calculated by multiplying the frequency score (0–5) by the severity score (0–5), thus ranging from 0–25. Among the 8 performance scores, the highest score was used to indicate the intensity level that was classified into 5 levels, very little for the highest performance score of 1–2, little for the highest performance score of 3–5, moderate for the highest performance score of 6–12, severe for the highest performance score of 15–16 and very severe for the highest performance score of 20–25 [12, 31]. In this study, we did not calculate the total OIDP score which was frequently used in literatures. Instead, the intensity was used as the main OIDP outcome because it was found to be the best outcome representing the severity of oral impacts perceived by the patients [31].

**Table 1:** Numbers of performances, performance score, extent and intensity of the original OIDP, OIDP-3, OIDP-2, OIDP frequency and OIDP severity

Index	Number of performances	Performance score	Extent	Intensity^b^
Original OIDP	8	Frequency score (0–5) x Severity score (0–5) (ranges from 0–25)	0–8 PWI^a^	Indicated by the highest performance score among the 8 performances [12, 31]
OIDP-3	3	Frequency score (0–5) x Severity score (0–5) (ranges from 0–25)	0–3 PWI^a^	Indicated by the highest performance score among the 3 performances
OIDP-2	2	Frequency score (0–5) x Severity score (0–5) (ranges from 0–25)	0–2 PWI^a^	Indicated by the highest performance score among the 2 performances
OIDP frequency	8	Frequency score (0–5)	0–8 PWI^a^	Indicated by the highest frequency score among the 8 performances
OIDP severity	8	Severity score (0–5)	0–8 PWI^a^	Indicated by the highest severity score among the 8 performances

^a^Performances with impacts

^b^classified into 5 level: very little, little, moderate, severe, very severe

Descriptive analyses on the demographic information, clinical characteristics of OLP, overall oral impacts, and impacts on 8 daily performances associated with OLP were performed. The shortened versions of the OIDP were developed in two manners, including only some performance items among the 8 items (OIDP-3 and OIDP-2) and either the frequency (OIDP frequency) or severity score (OIDP severity) (Table 1). For the inclusion of some items, the descriptive findings of oral impacts and the homogeneity among the 8 items of the OIDP index were considered. Items that were infrequently impacted by OLP were excluded; however, the retained items represented various dimensions, comprised physical, psychological, and social dimensions. The homogeneity or internal reliability analyses consisted of the standardized Cronbach’s alpha coefficient, Cronbach’s alpha if the item deleted, and the corrected item-total correlations. To determine which items could be excluded, a threshold of 0.8 standardized Cronbach’s alpha and correlation coefficients between 0.2–0.8 was applied [32]. For the shortened versions in which the outcomes were calculated from either the frequency score (OIDP frequency) or the severity score (OIDP severity), the highest frequency or severity score among the 8 frequency or severity scores was used to make the outcomes comparable to the intensity level of the oral impacts (Table 1).

The shortened versions should be as well associated with oral pain and clinical severity as the Thongprasom sign scores. The version should be able to discriminate between different clinical Thongprasom sign scores as the original OIDP did. The data were tested with the Kolmogorov Smirnov normality test and found to be non-parametrically distributed. Therefore, Spearman rank-order correlation coefficients were used to compare the shortened versions with the original OIDP and to determine the associations between the shortened and original OIDP and oral pain and clinical Thongprasom sign score. The discriminative ability of the shortened and original versions was identified by comparing the oral impact outcomes with those of the 1-step less clinically severe category of the Thongprasom sign score using the Mann–Whitney U Test. Significance defined as *P* < 0.05.

## Results

### Patient characteristics

The distribution of the patients’ characteristics is illustrated in Table 2. Out of 69 patients, ~ 80% were female. The mean age was 55.1 ± 13.9 years old (range 21–86 years old). The mean disease duration was 45.0 ± 49.6 months (range 1–264 months). Fifty out of 69 patients (72.5%) had one or more systemic conditions, whereas 19 patients (27.5%) had no systemic condition. The most common systemic disease found among the OLP patients was dyslipidemia (40.6%), followed by hypertension (39.1%) and diabetes mellitus (17.4%). About 7.2% of the OLP patients had heart diseases, thyroid diseases, or gastrointestinal diseases. Other systemic conditions were allergy (5.8%), anxiety/depression (2.9%), and 1.4% of the OLP patients had systemic lupus erythematosus, psoriasis, migraine, seborrheic dermatitis, or prostate gland disease.

**Table 2:** Distribution of the sociodemographic, clinical characteristics, and patient’s perception in OLP patients (*N* = 69)

Characteristics		N (%)
Sociodemographic		
Sex		
Female		55 (79.7)
Male		14 (20.3)
Age	Mean ± SD: 55.1 ± 13.9 years, range 21–86 years	
Systemic conditions	No	19 (27.5)
	Dyslipidemia	28 (40.6)
	Hypertension	27 (39.1)
	Diabetes mellitus	12 (17.4)
	Heart diseases	5 (7.2)
	Thyroid diseases	5 (7.2)
	Gastrointestinal diseases	5 (7.2)
	Allergy	4 (5.8)
	Anxiety/depression	2 (2.9)
	Systemic lupus erythematosus	1 (1.4)
	Psoriasis	1 (1.4)
	Migraine	1 (1.4)
	Seborrheic dermatitis	1 (1.4)
	Prostate gland disease	1 (1.4)
Clinical characteristics		
Disease duration	Mean ± SD: 45.0 ± 49.6 months, range 1–264 months	
OLP localization	Buccal mucosa	61 (88.4)
	Tongue	10 (14.5)
	Lip	10 (14.5)
	Gingiva	42 (60.9)
	Floor of the mouth	3 (4.3)
	Soft palate	2 (2.9)
Number of OLP affected sides^a^	1 affected side	5 (7.2)
	2 affected sides	28 (40.6)
	3 affected sides	13 (18.9)
	4 affected sides	15 (21.7)
	5 affected sides	4 (5.8)
	6 affected sides	4 (5.8)
OLP clinical severity (Thongprasom sign score)	Score 1	3 (4.4)
	Score 2	22 (31.9)
	Score 3	27 (39.1)
	Score 4	12 (17.4)
	Score 5	5 (7.2)
Patient’s perception		
Pain score^b^	Presence of pain	66 (95.6)
	Mean ± SD: 2.6 ± 2.3, range 0–8	
Overall impact (any performance)		67 (97.1)
Impact on daily performances	Eating	61 (88.4)
	Speaking	5 (7.2)
	Cleaning	45 (65.2)
	Sleeping and relaxing	4 (5.8)
	Emotional stability	43 (62.3)
	Smiling	10 (14.5)
	Working	6 (8.7)
	Social contact	12 (17.4)

^a^Maximum possible affected sides = 11 sides

^b^Maximum pain score = 10

The OLP clinical characteristics evaluation indicated that the buccal mucosa was the most prevalent (88.4%) affected site, followed by gingiva (60.9%), tongue and lip (14.5%), floor of the mouth (4.3%), and soft palate (2.9%). The most frequent number of OLP affected sides were two sides (40.6%), four sides (21.7%), and three sides (18.9%). The OLP clinical severity as classified by the Thongprasom sign score, based on the most severe lesion of each patient, was score 1 (4.4%), score 2 (31.9%), score 3 (39.1%), score 4 (17.4%), and score 5 (7.2%).

The pain perception results indicated that almost all of the patients (95.6%) complained of pain with a mean NRS pain score 2.6 ± 2.3 (range 0–8). The OHRQoL questionnaire revealed that the prevalence of the overall impacts of OLP to any performance was 97.1%.

### Characteristics of the oral impacts on daily performances

The percentages of patients experiencing oral impacts on each of the eight daily life performances are shown in Table 2. Most OLP patients experienced difficulties on three performances, Eating, Cleaning, and Emotional stability with percentages of 88.4%, 65.2%, and 62.3%, respectively. In contrast, oral impacts on the other five performances were reported by less than 20% of OLP patients, Speaking (7.2%), Sleeping and relaxing (5.8%), Smiling (14.5%), Working (8.7%), and Social contact (17.4%).

The extent and intensity of the oral impacts are displayed in Table 3. Among the 97.1% of patients having any kind of oral impact assessed by the original OIDP index, the majority (70%) reported affected impacts on 1–3 performances, while the remaining reported oral impacts on 4 performances (15.9%), 5 performances (5.8%), and 6–7 performances (2.9%). The intensity of oral impacts results demonstrated that 33.3% had an impact at a moderate intensity, followed by 18.9% with a severe and very severe intensity, and 13% with little and very little intensity.

**Table 3:** The extent and intensity of oral impacts assessed by the original OIDP, OIDP-3, and OIDP-2 and their associations with oral pain and clinical Thongprasom sign score (*N* = 69)

		N (%)		
		OIDP	OIDP-3^a^	OIDP-2^b^
Prevalence of impacts		97.1%	97.1%	94.2%
Extent of oral impacts (PWI)^c^				
0 performance		2 (2.9)	2 (2.9)	4 (5.8)
1 performance		16 (23.2)	16 (23.2)	26 (37.7)
2 performances		16 (23.2)	20 (29.0)	39 (56.5)
3 performances		16 (23.2)	31 (44.9)	-
4 performances		11 (15.9)	-	-
5 performances		4 (5.8)	-	-
6 performances		2 (2.9)	-	-
7 performances		2 (2.9)	-	-
8 performances		0	-	-
Intensity of oral impacts				
No		2 (2.9)	2 (2.9)	4 (5.8)
Very little		9 (13.0)	9 (13.0)	12 (17.4)
Little		9 (13.0)	10 (14.5)	9 (13.0)
Moderate		23 (33.3)	21 (30.4)	23 (33.4)
Severe		13 (18.9)	15 (21.8)	9 (13.0)
Very severe		13 (18.9)	12 (17.4)	12 (17.4)
Correlation coefficient with OIDP		-	r_s_ = 0.965	r_s_ = 0.911
*P* value			*P* < 0.001	*P* < 0.001
Pain^d^				
Correlation coefficient		r_s_ = 0.400	r_s_ = 0.372	r_s_ = 0.387
*P* value		*P* = 0.001	*P* = 0.002	*P* = 0.001
OLP clinical severity^d^				
Thongprasom sign score	N (%)			
Score 1	3 (4.4)	Severe	Severe	Severe
Score 2	22 (31.9)	Little^‡^	Little^‡^	Little^‡‡^
Score 3	27 (39.1)	Moderate^‡‡^	Moderate^‡‡^	Moderate^‡‡^
Score 4	12 (17.4)	Severe to Very severe^‡^	Severe^‡^	Severe^‡^
Score 5	5 (7.2)	Very severe	Very severe	Very severe
Correlation coefficient		r_s_ = 0.490	r_s_ = 0.461	r_s_ = 0.426
* P* value		*P* < 0.001	*P* < 0.001	*P* < 0.001

*r*_*s*_ Spearman rank-order correlation coefficient

^‡^*P* < 0.05

^‡‡^*P* < 0.01 (Mann–Whitney U test) compare with the 1-step less clinically severe category of the Thongprasom sign score

^a^Eating, Cleaning, Emotional stability

^b^Eating, Emotional stability

^c^Performances with impacts

^d^Associated with the intensity of oral impacts

The results described above revealed that three performances, Eating, Cleaning, and Emotional stability, were impacted in most OLP patients. Moreover, the analysis of the extent of impacts demonstrated that most OLP patients had impacts on 1–3 daily performances. Therefore, we developed a shortened version, OIDP-3, that consisted of the 3 performances (Eating, Cleaning, and Emotional stability). In addition, a shortened version, OIDP-2 that consisted of 2 performances (Eating and Emotional stability) was developed. Cleaning was excluded from OIDP-2 because Cleaning and Eating conceptually belong to the same dimension (physical dimension), whereas Emotional stability is conceptually considered a psychological dimension. The evaluation of the homogeneity of the OIDP indicated that the standardized Cronbach’s alpha coefficient was 0.81. The corrected item-total correlations ranged from 0.24 (Working) to 0.70 (Emotional stability) (Table 4). All eight items had correlation coefficients higher than the recommended threshold of 0.2 [32]. However, Cronbach’s alpha reduced to below the recommended threshold of 0.8 when Eating (0.74) or Emotional stability (0.73) was deleted. For the item of Cleaning, the deletion would slightly reduce Cronbach’s alpha to 0.79 which remained very close to the recommended threshold of 0.8. In addition, among the eight performances, Eating and Emotional stability had higher corrected item-total correlations compared with the others (0.66 and 0.70 respectively), while Cleaning revealed a lower corrected item-total correlation (0.44). These findings implied the importance of including Eating and Emotional stability in the index, and supported the conceptual possibility to exclude Cleaning from the index, resulting in the OIDP-2.

**Table 4:** Corrected item-total correlation, Cronbach’s alpha if item deleted from the OIDP index

Daily performances	Corrected item-total correlation	Cronbach’s alpha if item deleted^a^
Eating	0.66	0.74
Speaking	0.58	0.77
Cleaning	0.44	0.79
Sleeping and relaxing	0.56	0.76
Emotional stability	0.70	0.73
Smiling	0.51	0.76
Working	0.24	0.80
Social contact	0.54	0.77

Cronbach’s alpha (Standardized Items) of the original OIDP = 0.81

^a^Deletion of Eating or Emotional stability item reduced the Cronbach’s alpha to below a recommended threshold of 0.8 [32]. For other items, the deletion slightly reduced the Cronbach’s alpha

### Comparing OIDP-3 and OIDP-2 with the original OIDP

We calculated the extent and intensity levels of OIDP-3 and OIDP-2 (Table 3). OIDP-3 captured the 97.1% of patients having oral impacts assessed by the original OIDP. The patients who had oral impacts on more than 3 performances had impacts on Eating, Cleaning, and/or Emotional stability. Therefore, the performance of OIDP-3 in differentiating between patients with and without oral impacts was the same as that of the original OIDP. For OIDP-2, 97% of the patients having oral impacts assessed by the original OIDP were considered as having oral impacts (94.2% out of 97.1%). Thus, ~ 3% of patients with oral impacts would be excluded if OIDP-2 was used.

The OIDP-3 and OIDP-2 intensity distribution patterns were similar to that of the original OIDP. The highest proportion, approximately one-third of OLP patients, had oral impacts at the moderate level assessed by the original OIDP, OIDP-3, or OIDP-2. Comparing the proportions of patients with severe or very severe oral impacts, the 37.8% assessed by the original OIDP (18.9% with severe and 18.9% with very severe impacts) increased slightly to 39.2% when the OIDP-3 was used. However, this percentage markedly decreased to 30.4% when the OIDP-2 was used. These results implied that although very few patients with oral impacts would be excluded if OIDP-2 was used, they would consist of patients experiencing severe or very severe impacts. The overall associations between the intensity of the oral impacts assessed by the original OIDP and those of OIDP-3 and OIDP-2 were strongly significant (*P* < 0.001). The OIDP-3 obtained a very high correlation coefficient compared with the original OIDP (0.965), whereas, although the coefficient of OIDP-2 was slightly lower, it remained very high (0.911).

### Associations between OIDP, OIDP-3, and OIDP-2 and oral pain and clinical Thongprasom sign score

The association analyses of oral pain that indicated the criterion validity of the OIDP index revealed similar results for the three OIDP versions. The original OIDP and OIDP-2 were strongly significantly associated with oral pain (r_s_ = 0.400, *P* = 0.001 and r_s_ = 0.387, *P* = 0.001, respectively). Whereas, OIDP-3 obtained a slightly lower significance level (r_s_ = 0.372, *P* = 0.002) (Table 3).

The association analyses of the clinical Thongprasom sign score indicating the construct validity of the OIDP index are illustrated in Table 3. The intensity levels of the oral impacts assessed by the original OIDP increased step-wise in relation to the clinical severity of the Thongprasom sign score 2–5. The intensities of oral impacts were little, score 2; moderate, score 3; severe to very severe, score 4; and very severe, score 5. However, those assessed by OIDP-3 and OIDP-2 were almost the same as that of OIDP, except for score 4. Compared with the 1-step less clinically severe category of the Thongprasom sign score, there were significant differences in the intensity of the oral impacts assessed by the original OIDP, between score 1 and 2 (*P* < 0.05), score 2 and 3 (*P* < 0.01), and score 3 and 4 (*P* < 0.05). There was no significant difference between score 4 and 5. This pattern was also found for OIDP-3 and OIDP-2, revealing that the 2 shortened versions had the same discriminative ability as the original OIDP. The overall association between the clinical Thongprasom sign score and intensity of oral impacts assessed by the original OIDP (r_s_ = 0.490, *P* < 0.001) was similar to that of OIDP-3 (r_s_ = 0.461, *P* < 0.001) and OIDP-2 (r_s_ = 0.426, *P* < 0.001). However, three patients (4.4%) with Thongprasom sign score 1 reported a severe level of impacts, assessed by the original OIDP, OIDP-3, and OIDP-2, which was inconsistent with the pattern of positive associations between oral impacts and clinical severity.

### Associations between OIDP frequency and OIDP severity and the original OIDP, oral pain, and clinical Thongprasom sign score

The other shortened versions, OIDP frequency and OIDP severity, were associated with the original OIDP as well as the oral pain and clinical severity scores (Table 5). The OIDP frequency and OIDP severity scales were strongly significantly associated with the original OIDP (*P* < 0.001), however, the correlation coefficients of both versions (r_s_ = 0.768 and 0.880, respectively) were much lower than those of OIDP-3 and OIDP-2. Similarly, the associations with oral pain, although significant, had lower correlation coefficients and lower levels of significance (r_s_ = 0.354, *P* = 0.003 and r_s_ = 0.326, *P* = 0.006, respectively), compared with those of the original OIDP, OIDP-3, and OIDP-2.

**Table 5:** Associations between the intensity of oral impacts, the highest frequency score, and the highest severity score with oral pain and clinical Thongprasom sign score (*N* = 69)

		OIDP (intensity of impacts)	OIDP frequency (highest frequency score)	OIDP severity (highest severity score)
Correlation coefficient with OIDP		-	r_s_ = 0.768	r_s_ = 0.880
*P* value			*P* < 0.001	*P* < 0.001
Pain				
Correlation coefficient		r_s_ = 0.400	r_s_ = 0.354	r_s_ = 0.326
*P* value		*P* = 0.001	*P* = 0.003	*P* = 0.006
Thongprasom sign score	N (%)			
Score 1	3 (4.4)	Severe	3–4 times/week	Severe
Score 2	22 (31.9)	Little^‡^	1–2 times/week	Little^‡^
Score 3	27 (39.1)	Moderate^‡‡^	1–2 times/week	Moderate^‡‡^
Score 4	12 (17.4)	Severe to Very severe^‡^	Every day^‡^	Moderate to Severe
Score 5	5 (7.2)	Very severe	Every day	Severe
Correlation coefficient		r_s_ = 0.490	r_s_ = 0.365	r_s_ = 0.378
* P* value		*P* < 0.001	*P* = 0.002	*P* = 0.001

*r*_*s*_ Spearman rank-order correlation coefficient

^‡^*P* < 0.05, ^‡‡^*P* < 0.01 (Mann–Whitney U test) compare with the 1-step less clinically severe category of the Thongprasom sign score

Regarding the association with the clinical Thongprasom sign score, the medians of OIDP frequency were 3–4 times/week for Thongprasom sign score 1, 1–2 times/week for Thongprasom sign scores 2 and 3, and every day for Thongprasom sign scores 4 and 5. Although the frequency scores tended to increase with increasing clinical severity, no significant differences were detected compared with the 1-step less clinically severity score, except between score 3 and 4 (*P* < 0.05). For OIDP severity, the associations with Thongprasom sign scores were significant for 2 pairs, between scores 1 and 2 and scores 2 and 3. These findings indicated the lower discriminative ability of OIDP frequency and OIDP severity compared with the original OIDP, OIDP-3, and OIDP-2. The overall associations between OIDP frequency and OIDP severity and Thongprasom sign score were significant. However, the strengths of the associations and levels of significance for OIDP frequency (r_s_ = 0.365, *P* = 0.002) and OIDP severity (r_s_ = 0.378, *P* = 0.001) were lower than those of the original OIDP, OIDP-3, and OIDP-2.

## Discussion

Generally, OIDP data are collected through individual interview questionnaires. Some versions of the OIDP were modified to be self-administered questionnaires to reduce the burden on the interviewers and time consumption [33, 34]. Deciding on which mode of data collection would be appropriate also depends on the objective of using the index. The present study did not consider developing a self-administered version because our purpose was to develop patient-centered treatment services where the patients’ OHRQoL is taken into account. To achieve this, a positive relationship and trust between the dentist and patient are important. Patients should feel that they are respected, comfortable, and listened to about their problems. This is particularly important for older patients, including most OLP patients. Moreover, an interview elicits unarticulated thoughts, such as attitudes, feelings, and opinions, as well as patients’ desires [35]. Thus, we believe that the individual interview mode is the strength of the original OIDP index. Keeping the mode of individual interview unchanged, we, therefore, developed shortened versions of the OIDP and investigated their qualities compared with the original OIDP.

Our findings demonstrated that OIDP-3 and OIDP-2 might be alternatives to the original OIDP in measuring OHRQoL in OLP patients. Both versions’ findings were strongly associated with the original OIDP which established a convergent validity. Moreover, they demonstrated strong associations with oral pain and the clinical severity of OLP, as did the original OIDP, which confirmed their criterion and construct validity. Moreover, the discriminative ability of OIDP-3 and OIDP-2 was also similar to that of the original OIDP. Both shortened versions differentiated between different clinical severities assessed by the Thongprasom sign scoring system. However, OIDP-2 could not capture all patients with oral impacts assessed by the original OIDP. Although very few patients (~ 3%) were excluded, some of them had experienced severe oral impacts. Deciding whether this loss is acceptable or not might depend on the available resources. In case of limited time and personnel, interviews using OIDP-3 and OIDP-2 might be considered a practical option that could provide important information on oral impacts on daily performance. However, the original version of OIDP remains recommended to comprehensively understand OLP patients’ quality of life, if there are no chair time or interviewer constraints.

The present findings were consistent with previous studies investigating patients’ perception related to their OLP and other oral mucosal lesions, i.e., Eating, Cleaning, and Emotional stability were the most three frequently affected performances [24, 28, 36, 37]. A previous study that used the OIDP index found that Eating was the most often reported affected performance among patients with various types of oral mucosal lesions [24]. Consistent with our results, Emotional stability was the second most frequently reported and almost equal to that of Cleaning, whereas, Working and Social contact were the least impacted [24]. Furthermore, the highest prevalence of Eating and Emotional stability in the present study was consistent with those of an elderly Swedish population [26]. Likewise, studies using other OHRQoL indexes demonstrated that the majority of responses to the OHIP-14 in erosive/ulcerative OLP lesions were uncomfortable to eat, presence of a painful aching, followed by feeling less satisfied with life because of their oral health [6]. Moreover, Vilar-Villanueva et al. found that OLP patients with a worse OHRQoL gave higher OHIP scores in the dimensions of psychological discomfort and physical pain compared with the control group [38]. As mentioned above, these findings reaffirmed that the physical and psychological dimensions were the most affected in OLP patients. Therefore, Emotional stability was supported by earlier findings in which OLP patients had higher levels of stress, anxiety, and depression compared with healthy individuals [39–43]. The development of psychological change is thought to be influenced by issues regarding treatment limitations, side effects of some medications, frustration with the unpredictable nature of the lesions, and the potential for malignant transformation [9, 44, 45]. However, it is beyond the scope of the OIDP to identify detailed psychological issues. When further psychological assessment is required, validated tools, such as the Hospital Anxiety and Depression Scale, a screening test for anxiety and depression, and the 10-item Perceived Stress Scale are recommended as suggested by Wiriyakijja et al. [45]. Social dimension was not included in OIDP-3 and OIDP-2 because it is the consequence of the physical and psychological dimensions. Therefore, the use of only 2 dimensions, physical and psychological aspects, in OIDP-3 and OIDP-2 could be sufficient and better than a single measure, such as oral pain perception to complement OLP clinical measures and understand the patient’s problem related to OLP.

Our results demonstrated that OIDP frequency associated less strongly with oral pain and clinical severity compared with that of the original OIDP. Furthermore, OIDP frequency is unable to significantly discriminate between the clinical severities of OLP lesions. These results differ from Sulliman et al. who demonstrated the evaluative property of the OIDP frequency index of eight items [24]. The possible reasons for the discrepancy might be attributed to differences between the participants. Sulliman et al. assessed the OHRQoL impairment in dermatological patients who had different types of oral mucosal diseases, such as oral infections, vesiculo-bullous, and ulcerative lesions, as well as benign and malignant tumors. In addition, the varying nature of the clinical course between remission and exacerbation of OLP lesions might lead to an irregular pattern of oral impacts during a six-month period. Interestingly, typically OIDP severity demonstrated similar outcomes to that of the original OIDP, however, the difference was not significant. This finding agreed with those of Amilani et al. who demonstrated the promising psychometric properties of OIDP severity among adolescents in Sri Lanka [27]. However, the considerations for the use of a specific measure will vary depending on the specific objective. To fully assess the oral impacts on a patient’s life in a systematic way, 8 items covering all important daily performances in the physical, psychological, and social dimensions are needed. As discussed earlier, our shortened versions were purposed to be a quick tool for understanding the main oral impacts and evaluating the treatment response of OLP patients during the follow-up period.

Regarding the severity level of the oral impacts in three OLP patients with only white reticular lesions (score 1), this finding challenges the common belief that the reticular type of OLP is usually asymptomatic. This paradigm-challenging finding was supported by Adamo et al. and Vilar-Villanueva et al. in which patients with symptomatic reticular type reported higher levels of anxiety and depression compared with patients with asymptomatic reticular OLP [38, 46]. However, this finding should be interpreted with caution due to the small sample size of the reticular OLP group. Regarding other limitations in this study, statistical regression-based approaches to determine significance could not be performed due to the small sample size. Lastly, the sensitivity to change or the responsiveness to determine the minimal clinically important difference (MID) in relation to OLP treatment cannot be assessed in a cross-sectional study. Thus, it would be interesting to evaluate its use in a longitudinal study in OLP patients. If we could follow patients with OLP after treatment and assess their oral impacts relating to OLP when their OLP has changed, we would get more understanding on the association of OLP and oral impacts relating to OLP. Further studies using a larger sample size are recommended to confirm whether reticular lesions affect patients’ OHRQoL, and whether OIDP-3 or OIDP-2 is more appropriate for evaluating OHRQoL in OLP patients. Moreover, further studies to simply the OIDP or other OHRQoL indexes for using with patients having other dental problems, such as patients with tooth loss, would be very interesting. Although a simplified shortened version might lose some qualities as compared to a full original version, its practicality would make the integration of OHRQoL concept into dental practices possible.

## Conclusions

The present study demonstrates that OIDP-3, consisting of Eating, Cleaning, and Emotional stability, and OIDP-2, consisting of Eating and Emotional stability, performed similarly to the original OIDP in representing the degree of oral pain and clinical severity of the lesions. These shortened versions differentiated clinical differences as well as the original OIDP did. OIDP-3 captured all OLP patients with oral impacts assessed by the original OIDP, while very few cases were excluded if OIDP-2 was used. These findings might indicate the potential for their clinical use as a practical, quick tool for understanding patients and monitoring patient progress.

## Data Availability

All data generated or analysed during this study are included in this published article.

## Abbreviations

COMDQ: Chronic Oral Mucosal Disease Quality of Life index
CSS: Change in Symptom Scale
ICC: Intraclass correlation coefficient
MID: Minimal clinically important difference
NRS: Numeric Rating Scale
OHIP: Oral Health Impact Profile
OHRQoL: Oral Health-related Quality of life
OIDP: Oral Impact on Daily Performances
OLP: Oral lichen planus
r_s_: Spearman rank-order correlation coefficients
VAS: Visual Analogue Scale
